# Power spectral analysis of heart rate variability is useful as a screening tool for detecting sympathetic and parasympathetic nervous dysfunctions in Parkinson’s disease

**DOI:** 10.1186/s12883-022-02872-2

**Published:** 2022-09-10

**Authors:** Tomo Miyagi, Masanobu Yamazato, Takuto Nakamura, Takashi Tokashiki, Yukihiro Namihira, Kazuhito Kokuba, Satoshi Ishihara, Hirokuni Sakima, Yusuke Ohya

**Affiliations:** 1 Department of Cardiovascular Medicine, Nephrology and Neurology, Graduate School of Medicine, University of the Ryukyus, 207 Uehara, Nishihara-cho, Okinawa, 903-0215 Japan; 2 Division of Neurology, National Okinawa Hospital, National Hospital Organization, Okinawa, Japan

**Keywords:** Autonomic dysfunction, Coefficient of variation of R-R intervals (CVRR), ^123^I-metaiodobenzylguanidine (MIBG) scintigraphy, Non-motor symptom

## Abstract

**Background:**

Parkinson’s disease (PD) is a progressive neurodegenerative disorder that causes motor symptoms and autonomic dysfunction. However, autonomic function tests commonly performed in PD can only evaluate either the sympathetic or parasympathetic nervous system. Therefore, the purpose of this pilot study is to investigate whether power spectral analysis of heart rate variability could detect both sympathetic and parasympathetic nervous dysfunctions in patients with PD.

**Methods:**

Seventeen patients with PD and 11 healthy control subjects underwent electrocardiogram recording for the spectral analysis of heart rate variability to obtain values of low-frequency (LF) (0.04–0.15 Hz) and high-frequency (HF) (0.15–0.4 Hz) powers. Moreover, we examined the coefficient of variation of R–R intervals (CVRR) as a parameter of parasympathetic function in all participants and performed ^123^I-metaiodobenzylguanidine scintigraphy to measure the heart-to-mediastinum ratio as a parameter of cardiac sympathetic innervation in patients with PD.

**Results:**

The median age of control subjects and PD patients was 63 and 66 years old, respectively. The median Hoehn and Yahr scale of PD patients was stage 2. The values of resting LF and HF powers widely varied. The median values of resting LF powers of control subjects and PD patients and those of HF powers were 169 and 70 ms^2^, 279 and 65 ms^2^, respectively, the difference was statistically insignificant. Approximately 41% of patients with PD had values below the first quartile of resting LF powers (< 58 ms^2^) or HF powers (< 50 ms^2^); however, no control subject had such low values. Positive correlations were found between resting LF powers and heart-to-mediastinum ratios of ^123^I-metaiodobenzylguanidine uptake (*r* = 0.6) and between resting HF powers and CVRRs (*r* = 0.7). The resting LF power was also associated with CVRRs and constipation. Furthermore, a positive correlation was observed between resting LF powers and resting HF powers in patients with PD (*r* = 0.8).

**Conclusions:**

The power spectral analysis of heart rate variability may be useful as a screening tool for detecting autonomic dysfunctions by detecting low resting LF and HF powers in patients with PD. Sympathetic and parasympathetic nerves may be concurrently damaged in patients with PD.

**Supplementary Information:**

The online version contains supplementary material available at 10.1186/s12883-022-02872-2.

## Background

Parkinson’s disease (PD) is a neurodegenerative disease with bradykinesia, muscle rigidity, and resting tremor as the three major signs. In addition to the motor symptoms, various non-motor symptoms, such as psychiatric symptoms, autonomic dysfunction, olfactory dysfunction, and cognitive dysfunction, appear in patients with PD. Non-motor symptoms are thought to affect the quality of life [1]. Autonomic neuropathy may cause gastrointestinal symptoms, urination disorders, orthostatic hypotension, sexual dysfunction, and other autonomic symptoms. These symptoms are thought to be a result of damage to the sympathetic and parasympathetic nervous systems in patients with PD. Pathologically, it has been reported that α-synuclein is deposited in the sympathetic and parasympathetic nervous systems in patients with PD [2–5].

The autonomic function tests commonly performed in patients with PD are ^123^I-metaiodobenzylguanidine (MIBG) myocardial scintigraphy and the coefficient of variation of R–R interval (CVRR). ^123^I-MIBG myocardial scintigraphy could evaluate cardiac sympathetic nerve activity and is performed to detect sympathetic nerve disorders in patients with neurodegenerative diseases. A decrease in the cardiac uptake of ^123^I-MIBG [6, 7] is suggestive of a disorder of cardiac sympathetic postganglionic fibers. Evaluating damage to the postganglionic fibers of the cardiac sympathetic ganglia is possible using this test, thus making it as the preferred tool as an adjunct diagnosis for patients with PD in the early phase [8]. However, it is invasive and expensive, and only a limited number of facilities can perform this examination. The CVRR has been used to evaluate the function of parasympathetic nerves. A decrease in the CVRR indicates dysfunction of the parasympathetic nervous system [9, 10]. However, these autonomic function tests can only evaluate either the sympathetic or parasympathetic nervous system.

The power spectral analysis of heart rate variability (HRV) is a noninvasive and easy examination, which can determine the low-frequency (LF) and high-frequency (HF) powers of HRV. The LF power is primarily affected by sympathetic nervous activity and also by baroreceptor reflexes [11], and a correlation was found between LF power and muscle sympathetic nerve activity in healthy subjects [12]. The HF power is affected by respiratory fluctuations [11] and is increased by vagal stimulation and reduced by inhibiting muscarinic receptors, or vagal blockade, suggesting that the HF power reflects parasympathetic activities [13]. Some studies have evaluated autonomic function in patients with heart failure or diabetes mellitus using spectral analysis of HRV. Only the LF power decreased in patients with severe heart failure [14], whereas both the LF and HF powers decreased in patients with diabetes mellitus [15]. Therefore, the spectral analysis of HRV can evaluate both sympathetic and parasympathetic nerve activities separately in the same examination and may detect different patterns of autonomic dysfunction depending on the disease. Despite these advantages, the power spectral analysis of HRV is an indirect examination of autonomic nervous system function, and the accuracy of detecting autonomic neuropathy is inferior to that of ^123^I-MIBG myocardial scintigraphy that directly visualizes sympathetic nerve endings [16]. Therefore, the power spectral analysis of HRV is not currently used as an autonomic function test for patients with PD as a substitute to scintigraphy.

Although both sympathetic and parasympathetic nerves are impaired in patients with PD, the degree and characteristics of autonomic neuropathy cannot be fully evaluated using existing examinations. Although the power spectral analysis of HRV could separately evaluate both sympathetic and parasympathetic nerve dysfunctions in the same examination and is easy to perform, the usefulness of this examination as a screening test before performing ^123^I-MIBG myocardial scintigraphy in patients with PD has not been well elucidated. Therefore, we conducted this pilot study to examine whether the spectral analysis of HRV could detect both sympathetic and parasympathetic nerve dysfunctions in patients with PD.

## Methods

### Participants

In this study, 19 patients with PD and 11 healthy volunteers were enrolled. The patients with PD who participated in this study were outpatients of either three neurologists working at the University of the Ryukyus Hospital from March 2016 to October 2017. A clinical diagnosis of idiopathic PD was based on the Movement Disorder Society Clinical Diagnostic Criteria for PD (2015) [8]. The patients were prescribed regular antiparkinsonian drugs, such as levodopa and carbidopa or benserazide, amantadine, monoamine oxidase B inhibitor, and dopamine agonists. The clinical stage of the patients was assessed using the Hoehn and Yahr (H&Y) Scale and the United Parkinson’s Disease Rating Scale motor score (UPDRS Parts 2 and 3). The control subjects were healthy volunteers aged 50–70 years, who had not been diagnosed with PD. Constipation was defined as a participant who had no bowel movements daily or was taking laxatives. We excluded patients we could not be assessed due to arrhythmia, those who could not stay calm, those with dementia, those with symptomatic orthostatic hypotension, and those with other autonomic failures, such as diabetes mellitus, from this study. The study was approved by the Ethics Committee of the University of the Ryukyus (#710) according to the revised Declaration of Helsinki. Written informed consent to participate in this study was obtained from all participants.

### Examinations

#### Spectral analysis of HRV and head-up tilt test

The power spectral analysis of HRV analyzed with the maximum entropy method was performed on all participants using Kiritsu Meijin of CROSSWELL Co., Ltd. The examination was conducted between 9 and 10 AM in fasting conditions. First, all participants rested on the tilt table in the supine position for 30 min. After resting, electrocardiogram and blood pressure measurements were performed on the participants every minute for 3 min in the supine position, and then, the tilt table angle was set to 90° for passive standing for another 3 min. Spectral analysis of HRV was performed to obtain values of LF (0.04–0.15 Hz) power, HF (0.15–0.4 Hz) power, and LF/HF ratios. Orthostatic hypotension was diagnosed when the systolic blood pressure decreased by 20 mmHg or more and/or diastolic blood pressure decreased by 10 mmHg or more within 3 min after standing up.

#### ^123^I-MIBG myocardial scintigraphy and CVRR

In the PD group, the CVRR was measured and ^123^I-MIBG myocardial scintigraphy was performed 1 month before or after the power spectral analysis of HRV was examined. In the healthy control group, the CVRR was measured on the same day as the spectral analysis of HRV. ^123^I-MIBG myocardial scintigraphy was performed as follows: planar scintigraphic images in the anterior view were captured by a single-head gamma camera (INFINIA Hawkeye4, GE Healthcare Japan) 15 min (early) and 4 h (delayed) after the intravenous injection of MIBG (111 MBq, FUJIFILM Toyama Chemical Co., Ltd). The normal range of H/M ratio for MIBG myocardial scintigraphy was 2.2 or higher in our institution. The CVRR was measured as follows: the participants had a rest in the supine position for 30 min and underwent electrocardiogram for 3 min at rest and for 1 min with deep breathing. The CVRR of 2.0 or less was considered to be parasympathetic dysfunction in the current study [17].

### Statistical analysis

All analyses were performed using JMP® 14 (SAS Institute Inc., Cary, NC, USA). Group comparisons were made using Student’s t-test, Pearson’s chi-square test, and Wilcoxon’s rank-sum test, and correlations were examined using simple regression analysis. The threshold for statistical significance was set at a *p*-value of less than 0.05.

## Results

### Characteristics of participants

In this study, 19 patients with PD and 11 healthy control subjects were enrolled. Two patients with PD were excluded from the study due to a change in diagnosis and severe resting tremor that affected electrocardiogram recording. Table 1 shows the clinical characteristics of the healthy controls and patients with PD and the results of the autonomic function tests. No significant differences in age, gender, resting blood pressure, and heart rate were observed between the healthy controls and patients with PD. In patients with PD, the average duration of illness and scores for the UPDRS parts 2 and 3 were 33.5 months, 10.0, and 17.7 points, respectively. The median H&Y was stage 2, and the average levodopa equivalent daily dose (LEDD) was 386 mg/day. Constipation was found in 76% of patients with PD, which was significantly higher than the percentage of healthy controls with constipation. The deep breathing CVRR was significantly lower in patients with PD than that in healthy controls. The median values of resting LF and HF powers in the supine position tended to be lower in patients with PD than those in healthy controls; however, the difference was statistically insignificant. Orthostatic hypotension was observed in six patients with PD (35%), and the prevalence of orthostatic hypotension was significantly higher in patients with PD than that in healthy controls. In passive standing, healthy control subjects showed significantly decreased HF power values *(p* < *0.05*) but not in patients with PD (*p* = *0.33*). The values of LF power and the LF/HF ratio did not change during passive standing in both groups. Blood pressure, heart rate, and values of LF and HF powers during head-up tilt test of representative control subject and patient with PD with orthostatic hypotension are shown in Supplementary Fig. 1.

**Table 1:** Characteristics and the results of autonomic function tests in healthy control subjects and patients with Parkinson’s disease (PD)

	Controls	PD	*P*-value
Number	11	17	
Age (year)	63 (59–64)	66 (60–72)	0.138
Female (%)	55	47	0.700
Systolic BP (mmHg)	125 ± 14	129 ± 20	0.580
Diastolic BP (mmHg)	75 ± 8	76 ± 11	0.768
Heart rate (beats/min)	64 ± 11	64 ± 9	0.956
Duration of disease (months)	-	33.5 ± 7.2	
UPDRS Part 2 (points)	-	10.0 ± 1.4	
UPDRS Part 3 (points)	-	17.7 ± 2.3	
H&Y stage	-	2.0 (1.5–3.0)	
LEDD (mg/day)	-	386 ± 279	
Constipation (%)	9	76	< 0.001
CVRR, rest (%)	3.7 (1.8–5.9)	2.6 (1.7–4.0)	0.359
CVRR, deep breath (%)	4.4 (3.9–6.8)	2.8 (2.1–4.0)	0.018
Early H/M ratio	-	1.92 ± 0.56	
Delayed H/M ratio	-	1.69 ± 0.71	
Orthostatic hypotension (%)	0	35	0.026
LF power, supine (ms^2^)	169 (126–427)	70 (47–307)	0.086
HF power, supine (ms^2^)	279 (85–342)	65 (27–302)	0.070
LF/HF ratio, supine	1.22 (0.78–3.33)	1.47 (0.72–2.76)	0.814
LF power, standing (ms^2^)	125 (65–542)	84 (41–198)	0.115
HF power, standing (ms^2^)	62 (43–82) *	40 (15–127)	0.589
LF/HF ratio, standing	3.74 (1.87–9.4)	2.52 (0.86–7.74)	0.438

Values are expressed as the mean ± SD or median (25%–75% quartile)

Constipation: Participants who had no bowel movements daily or were taking laxatives

*Abbreviations: UPDRS* Unified Parkinson’s disease rating scale, *H&Y* Hoehn and Yahr, *LEDD* Levodopa equivalent daily dose, *LF* Low frequency, *HF* High frequency, *CVRR* Coefficient of variation of RR intervals, *BP* Blood pressure

^*^p < 0.05 vs respective powers of supine position, paired t-test

### The distribution of resting LF powers and resting HF powers in healthy control subjects and patients with PD

**Fig. 1:**
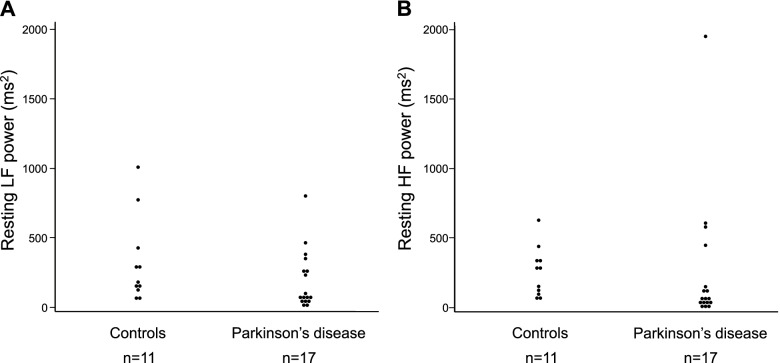
The distribution of resting LF power (**A**) and resting HF power (**B**) in control subjects and Parkinson's disease (PD). **A** The values of LF powers of all participants varied from 12 ms^2^ to 1,008 ms^2^. The median values of resting LF power were 169 ms^2^ in control subjects and 70 ms^2^ in patients with PD. The difference was insignificant in the Wilcoxon rank-sum test (*p* = 0.09). **B** The values of HF powers of all participants varied from 7 ms^2^ to 1,957 ms^2^. The median values of resting HF power were 279 ms^2^ in control subjects and 65 ms^2^ in the patients with PD. The difference was insignificant in the Wilcoxon rank-sum test (*p* = 0.07). Focusing on the very low values of resting LF and HF powers, approximately 41% of PD patients had values below the first quartile of resting LF powers (< 58 ms^2^) or HF powers (< 50 ms^2^); however, no control subject had such low values. Abbreviations: LF, low frequency; HF, high frequency

The values of LF powers (Fig. 1A) and HF powers (Fig. 1B) of all participants widely varied from 12 ms^2^ to 1,008 ms^2^ and from 7 ms^2^ to 1,957 ms^2^, respectively. The median values of resting LF power were 169 ms^2^ in control subjects and 70 ms^2^ in patients with PD. The median values of resting HF power were 279 ms^2^ in control subjects and 65 ms^2^ in the patients with PD. No difference was found between the control subjects and patients with PD in the Wilcoxon rank-sum test (LF: *p* = 0.09, HF: *p* = 0.07). However, when we focused on the very low values of resting LF powers and HF powers, approximately 41% of PD patients had values below the first quartile of resting LF powers (< 58 ms^2^) or HF powers (< 50 ms^2^); however, no control subject had such low values.

### Correlation between values of resting LF or HF powers and values of conventional autonomic function tests

**Fig. 2:**
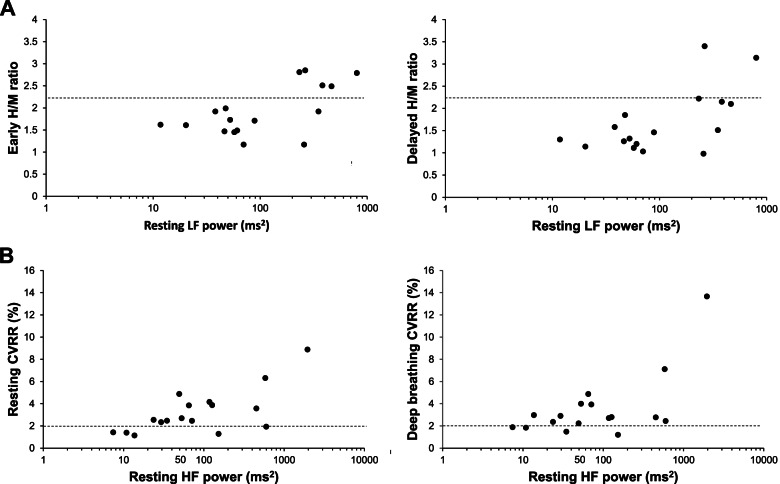
Correlations between resting LF power and the heart-to-mediastinum (H/M) ratio of ^123^I-meta-iodobenzylguamidine (MIBG) uptake (**A**) and correlations between resting HF power and the coefficient variation of R–R intervals (CVRR) (**B**) in patients with PD. **A** Significant correlations were found between the H/M ratio and resting LF power (early, *r* = 0.57, *p* < 0.05; delayed, *r* = 0.62, *p* < 0.05). The dashed line shows the cutoff value for the H/M ratio (2.2). **B** Significant correlations were found between the CVRR and resting HF power (rest, *r* = 0.66, *p* < 0.05; deep breath, *r* = 0.57, *p* < 0.05). The dashed line shows the cutoff value for the CVRR (2.0)

To examine whether the power spectral analysis of HRV would be associated with conventional autonomic function tests, we examined the correlations between values of resting LF power and the heart-to-mediastinum (H/M) ratio of ^123^I-MIBG uptake, a parameter of sympathetic innervation, and between values of resting HF power and values of the CVRR, a parameter of parasympathetic function. A significant correlation was observed between values of resting LF power and the H/M ratio of the early and delayed phases of ^123^I-MIBG uptake (early, *r* = 0.57, *p* < 0.05; delayed, *r* = 0.62, *p* < 0.05) in patients with PD (Fig. 2A). The resting LF power was not associated with presence of orthostatic hypotension in patients with PD (*p* = *0.76*). In healthy control subjects, the values of resting HF power and resting CVRR tended to be correlated, however, the correlation was statistically insignificant (*p* = *0.09*); no correlation was found between values of resting HF power and deep-breathing CVRR (*p* = *0.21*). In patients with PD, significant correlations were observed between values of resting HF power and resting (*r* = 0.66, *p* < 0.05) and deep breathing CVRR (*r* = 0.57, *p* < 0.05) (Fig. 2B). We could not find the association between resting HF power and constipation (*p* = *0.37*) in patients with PD. Regarding the LF power and parasympathetic function, there was a significant correlation between values of resting LF power and values of CVRRs (resting, *p* < *0.05*; deep breathing, *p* < *0.05*) and a significant association was found between values of resting LF power and constipation (*p* < *0.05*) in patients with PD.

Furthermore, we performed a multiple regression analysis since there was a significant negative correlation was observed between resting LF (*p* < *0.05*) or HF power (*p* < *0.05*) and age in patients with PD. The multiple regression analysis revealed that the correlation of LF and HF power with H/M ratio of the early phase of cardiac MIBG uptake (*R*^2^ = 0.68, β = 0.54, *p* < *0.01*) and CVRR (*R*^2^ = 0.54, β = 0.50, *p* < *0.01*) was still independently significant after controlling for age. We also performed subgroup analysis and found that only women but not men with patients with PD had a significant association of LF and HF power with cardiac MIBG uptake and CVRRs and resting LF power with resting HF power (Supplementary Table 1, Supplementary Fig. 2–4). Ten of 12 patients with PD with an H/M ratio of less than 2.2 had resting LF powers of less than 100 ms^2^. Three of five patients with PD with resting CVRR of less than 2.0% had resting HF power of less than 50 ms^2^. Furthermore, patients with PD with an H/M ratio of less than 2.2 had significantly lower values of resting CVRR than those with an early H/M ratio of more than 2.2 (2.3% ± 0.9% vs. 5.6% ± 2.1%, *p* < *0.01*), and patients with PD with resting CVRR below 2.0% had an H/M ratio of less than 2.2.

### Correlation between values of resting LF and HF powers in patients with PD

**Fig. 3:**
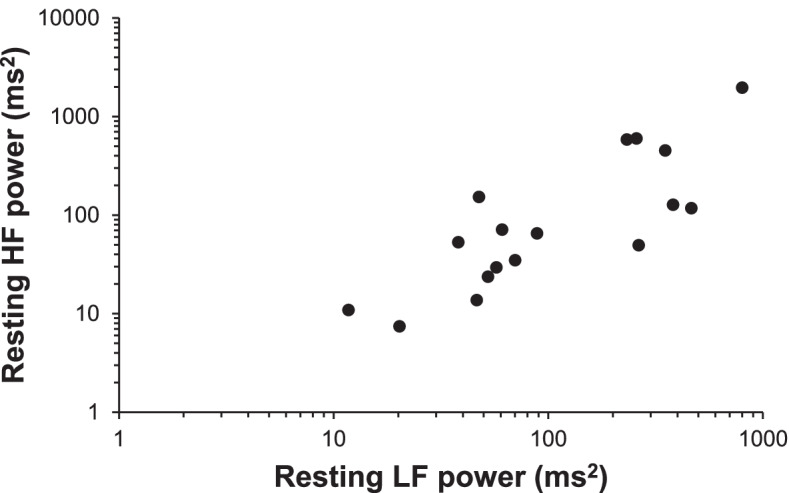
The correlation between values of resting LF and HF powers in patients with PD. A positive correlation was observed between values of resting LF and HF powers (*r* = 0.82, *p* < 0.01)

Figure 3 shows the correlation between values of resting LF and HF powers in patients with PD. A significant positive correlation was observed between values of resting LF and HF powers (*r* = 0.82, *p* < 0.01). No significant correlations were observed between UPDRS Part 2 and 3 scores, illness duration, LEDD, orthostatic hypotension, and values of resting LF or HF powers. The correlation of LF power and HF power was still independently significant after controlling for age (*R*^2^ = 0.37, β = 0.61, *p* < *0.01*).

## Discussion

In this study, we investigated whether the spectral analysis of HRV could detect both sympathetic and parasympathetic nervous dysfunctions in patients with PD. The low values that were below the first quartile of resting LF or HF powers were observed in the patients with PD but not in the healthy control subjects. Positive correlations were observed between the values of resting LF power and the H/M ratio of ^123^I-MIBG uptake and between the values of resting HF power and CVRR. These results suggested that the power spectral analysis of HRV may be useful as a screening tool for detecting autonomic dysfunctions in patients with PD by detecting low resting LF and HF power values.

Studies have reported decreased accumulation in the myocardial ^123^I-MIBG uptake in patients with PD [6, 7], and myocardial scintigraphy could detect impaired cardiac sympathetic postganglionic fibers. A study of subjects not limited to PD has found a correlation between myocardial MIBG uptake and LF power [18]. When restricted to patients with PD, some reports have shown no correlation between ^123^I-MIBG myocardial scintigraphy and the power spectral analysis of HRV [19–21]. In this study, the values of resting LF power were positively correlated with the H/M ratio of ^123^I-MIBG uptake in patients with PD (Fig. 2A). This may be because the values of LF power in patients with PD who participated in this study were widely distributed from extremely low values to values similar to those in healthy subjects (Fig. 1). Furthermore, the CVRR is an examination that could evaluate the function of the parasympathetic nervous system [9, 10]. Some studies have reported decreased CVRR in patients with PD [22, 23]. However, few studies have examined the relationship between CVRR and the spectral analysis of HRV. In this study, the values of resting HF power were positively correlated with resting or deep breathing CVRR in patients with PD (Fig. 2B). Therefore, these results suggest that the values of resting LF and HF powers reflect the degree of resting sympathetic and parasympathetic activities, respectively, in patients with PD. However, LF power of HRV also associated with parasympathetic activity in the present study. We do not know the weight of contribution of parasympathetic activity to the value of LF power in the present stage. We may need to use more specific non-invasive examination such as LF power of blood pressure variability to evaluate sympathetic activity in the future study.

Studies have suggested a decrease in LF and HF powers in patients with PD compared with those in healthy control subjects [19, 20, 24–26]. Katagiri A et al. have reported a reduction in LF and HF components in patients with PD compared with those in control subjects [20]. In the report, the mean values of the LF and HF components in patients with PD were 99.5 ms^2^ and 79.3 ms^2^, respectively. In this study, the median values of resting LF and HF powers in the supine position in patients with PD were not significantly lower than those in healthy controls (Fig. 1). However, we found that the low values that were below the first quartile of resting LF or HF powers were observed only in the patients with PD in this study. We hypothesized that the low values of resting LF and HF powers, which were found only in patients with PD and not in healthy controls, indicated autonomic dysfunction.

Furthermore, whether low values of resting LF or HF power were related to obvious sympathetic or parasympathetic dysfunction as determined using conventional examination methods was investigated. Ten of 12 patients with PD with impaired cardiac sympathetic postganglionic fibers as determined by myocardial scintigraphy had values of resting LF power of less than 100 ms^2^ (Fig. 2A). Three of five patients with PD with parasympathetic dysfunction as determined by the CVRR had values of resting HF power of less than 50 ms^2^ (Fig. 2B). These results suggested that the power spectral analysis of HRV may be useful in detecting cardiac sympathetic and parasympathetic dysfunctions by detecting low resting LF and HF power values. Although the number of participants in this study is relatively small, we propose that resting LF power values of less than 100 ms^2^ and resting HF power values of less than 50 ms^2^ could be used to screen for sympathetic and parasympathetic dysfunction, respectively.

The spectral analysis of HRV may be able to evaluate the reflex function of the sympathetic and parasympathetic nervous systems by applying a physiological load or pharmacological intervention [13]. In this study, passive standing significantly decreased the values of HF powers in healthy controls, whereas the HF powers did not change in patients with PD (Table 1), suggesting that the reflex function of the parasympathetic nervous system was maintained in control subjects; however, in patients with PD, the reflex function of the parasympathetic nervous system was impaired. Orthostatic hypotension was not observed in the healthy controls in this study, suggesting that their baroreflex functions were maintained; however, the LF power did not change during standing in healthy controls, suggesting that this examination needs other interventions to evaluate the reflex function of the sympathetic nervous system in our participants.

Orthostatic hypotension is important as a sympathetic dysfunction and cardiac sympathetic denervation. However, in the present study, we could not find the association between resting LF power and orthostatic hypotension in patients with PD. We speculated that other factors such as volume status and psychological factors might be contributed the result. Furthermore, constipation is an important symptom as parasympathetic dysfunction. However, we did not find any association between constipation and resting HF power. Since parasympathetic innervation of cardiovascular system (vagal nerve) and colorectal system (pelvic nerve) is different so we speculated that this difference may have contributed to the results.

In this study, a positive correlation was observed between values of resting LF and HF powers in patients with PD (Fig. 3). It has been reported that the H/M ratio correlates with the CVRR in early PD [23]. This suggests that cardiac sympathetic and parasympathetic dysfunctions occur alongside each other. Pathologically, Lewy bodies and loss of neural cells has been observed in the sympathetic postganglionic fibers, sympathetic ganglia, thoracic intermediolateral nucleus, cardiac plexus, the nucleus of the solitary tract, the rostral ventrolateral medulla, and the dorsal motor nucleus of the vagus nerve in patients with PD [2, 4, 5]. Furthermore, Iwanaga et al. have reported that Lewy bodies were present in both tyrosine hydroxylase-positive and tyrosine hydroxylase-negative nerve processes in the cardiac plexus of patients with PD [27]. These pieces of evidence suggested that PD causes sympathetic and parasympathetic nerve disorders both centrally and peripherally. In fact, in this study, sympathetic dysfunction was observed in patients with PD who had parasympathetic dysfunction. Our observation that resting LF and HF powers are positively correlated with each other suggests that the sympathetic and parasympathetic nervous systems are concurrently damaged in patients with PD.

In this study, 53% of patients with PD had relatively mild motor symptoms with H&Y less than stage 2; however, the proportion of patients with PD with low resting LF and HF power values was higher than that of healthy subjects with low resting LF and HF power values. In other words, autonomic dysfunction may have progressively developed before the appearance of overt motor symptoms. It has been reported that non-motor symptoms in PD can precede onset of motor symptoms [28, 29]. In addition, the H/M ratio and Scales for Outcomes in Parkinson’s Disease-Autonomic have been shown to decrease in proportion to disease duration in patients with PD [30, 31]. Based on previous reports that autonomic dysfunction precedes motor symptoms, the spectral analysis of HRV may be useful for auxiliary diagnosis before performing ^123^I-MIBG myocardial scintigraphy.

In this study, we analyzed separately with gender and find that only women but not men with PD had a significant association of LF and HF power with cardiac MIBG uptake and CVRR and resting LF power with resting HF power (Supplementary Table 1, Supplementary Fig. 2–4). However, we considered that gender difference of these results was inconclusive, since our sample size was small and there were several differences between men and women in baseline characteristics such as LEDD and median resting LF power. We may need to increase in number of patients with PD to evaluate subgroup analysis such as gender difference of power spectral analysis of HRV.

This study has several limitations. First, the number of study participants was relatively small, and the study was conducted in a single facility. Second, since this is a cross-sectional study, it does not represent the pattern of changes of autonomic dysfunction in individual patients based on disease progression. Furthermore, we could not mention the relationship between motor symptoms and autonomic dysfunction because we could not recruit patients according to the severity of their motor symptoms. Third, since we did not discontinue antiparkinsonian drugs, including monoamine oxidase B inhibitors, we could not exclude the effects of the drugs. On autopsy, some patients diagnosed with clinical PD may be pathologically proven to have progressive supranuclear palsy, corticobasal syndrome, or multiple system atrophy. Therefore, some patients might have been misdiagnosed.

In conclusion, the power spectral analysis of HRV could be useful as a screening tool for detecting sympathetic and parasympathetic nervous dysfunctions in patients with PD in the early phase, and the sympathetic and parasympathetic nerves are concurrently damaged in patients with PD.

## Supplementary Information

**Additional file 1: Supplementary Figure 1.** Blood pressure, heart rate, and values of LF and HF powers during head-up tilt test of representative control subject (*A*) and Parkinson’s disease with orthostatic hypotension (*B*). **Supplementary Table 1.** Characteristics and the results of autonomic function tests in healthy control subjects and patients with Parkinson’s disease (PD) by gender. **Supplementary Figure 2.** Correlations between resting LF power and heart-to-mediastinum (H/M) ratio of ^123^I-meta-iodobenzylguamidine (MIBG) uptake (*A*) and correlations between resting HF power and coefficient variation of RR intervals (CVRR) (*B*) in male patients with PD. **Supplementary Figure 3.** Correlations between resting LF power and heart-to-mediastinum (H/M) ratio of ^123^I-meta-iodobenzylguamidine (MIBG) uptake (*A*) and correlations between resting HF power and coefficient variation of RR intervals (CVRR) (*B*) in female patients with PD. **Supplement Figure 4.** The correlation between resting LF power and HF power in the patients with PD by gender (*A.* Men, *B. *Women).

## Data Availability

The datasets used and/or analysed during the current study are available from the corresponding author on reasonable request.

## Abbreviations

CVRR: Coefficient of variation of R–R intervals
HRV: Heart rate variability
H/M: Heart-to-mediastinum
HF: High-frequency
H&Y: Hoehn and Yahr
LEDD: Levodopa equivalent daily dose
LF: Low-frequency
MIBG: Metaiodobenzylguanidine
PD: Parkinson’s disease
UPDRS: United Parkinson’s Disease Rating Scale motor score
