# Supplementary material for: Power spectral analysis of heart rate variability is useful as a screening tool for detecting sympathetic and parasympathetic nervous dysfunctions in Parkinson’s disease
Source: BMC Neurol. 2022 Sep 10;22:339. doi: 10.1186/s12883-022-02872-2 (PMC9463782; doi:10.1186/s12883-022-02872-2)
Supplement: Supplementary file 1 — Additional file 1: Supplementary Figure 1. Blood pressure, heart rate, and values of LF and HF powers during head-up tilt test of representative control subject (A) and Parkinson’s disease with orthostatic hypotension (B). Supplementary Table 1. Characteristics and the results of autonomic function tests in healthy control subjects and patients with Parkinson’s disease (PD) by gender. Supplementary Figure 2. Correlations between resting LF power and heart-to-mediastinum (H/M) ratio of 123I-meta-iodobenzylguamidine (MIBG) uptake (A) and correlations between resting HF power and coefficient variation of RR intervals (CVRR) (B) in male patients with PD. Supplementary Figure 3. Correlations between resting LF power and heart-to-mediastinum (H/M) ratio of 123I-meta-iodobenzylguamidine (MIBG) uptake (A) and correlations between resting HF power and coefficient variation of RR intervals (CVRR) (B) in female patients with PD. Supplement Figure 4. The correlation between resting LF power and HF power in the patients with PD by gender (A. Men, B. Women). [file 12883_2022_2872_MOESM1_ESM.docx]

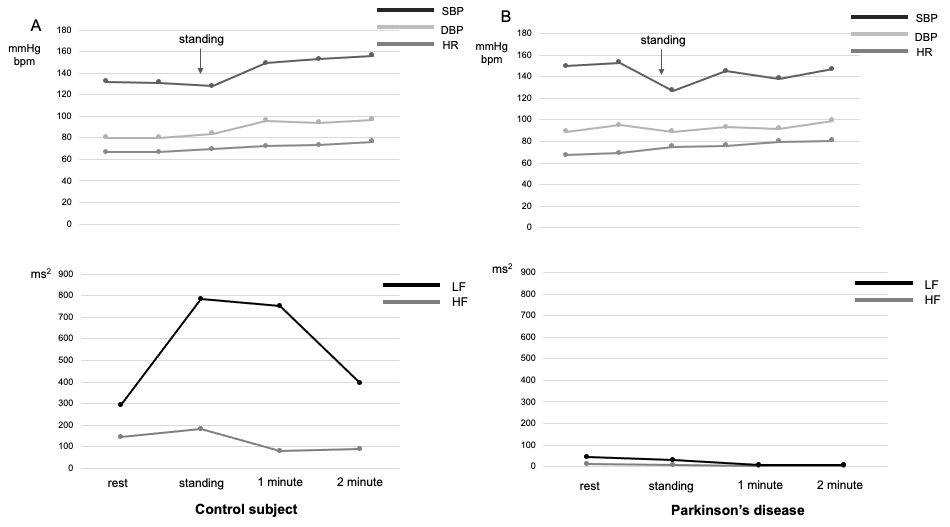


Supplementary Figure 1. Blood pressure, heart rate, and values of LF and HF powers during head-up tilt test of representative control subject (***A***) and Parkinson's disease with orthostatic hypotension (***B***).

Abbreviations: LF, low frequency; HF, high frequency; SBP, systolic blood pressure; DBP, diastolic blood; HR, heart rate

Supplementary Table 1. Characteristics and the results of autonomic function tests in healthy control subjects and patients with Parkinson’s disease (PD) by gender

Control subjects

| **ｖ** | **Men** | **Women** | ***p*-value** |
| --- | --- | --- | --- |
| Number | 5 | 6 |  |
| Age (year) | 62 (60–67) | 63 (52–64) | 0.647 |
| Systolic BP (mmHg) | 125 ± 13 | 124 ± 16 | 0.906 |
| Diastolic BP (mmHg) | 73 ± 4 | 77 ± 10 | 0.477 |
| Heart rate (beats/min) | 61 ± 14 | 66 ± 9 | 0.498 |
| Constipation (%) | 0 | 100 | 0.338 |
| CVRR, rest (%) | 4.8 (2.7–7.3) | 2.7 (1.8–4.3) | 0.235 |
| CVRR, deep breath (%) | 5.6 (3.1–7.5) | 4.2 (3.8–6.4) | 0.523 |
| Orthostatic hypotension (%) | 0 | 0 |  |
| LF power, supine (ms^2^) | 293 (121–718) | 142 (109–410) | 0.315 |
| HF power, supine (ms^2^) | 279 (115 –318) | 228 (72–490) | 1.00 |
| LF/HF ratio, supine | 3.33 (0.65–5.4) | 1.04 (0.71–1.61) | 0.411 |
| LF power, standing (ms^2^) | 327 (120–647) | 79 (57–306) | 0.171 |
| HF power, standing (ms^2^) | 65 (53–83) | 54 (35–76) | 0.315 |
| LF/HF ratio, standing | 4.35 (2.4–13.7) | 2.98 (1.8–4.3) | 0.315 |

Patients with PD

| **ｖ** | **Men** | **Women** | ***p*-value** |
| --- | --- | --- | --- |
| Number | 9 | 8 |  |
| Age (year) | 63 (60–66) | 70 (60–75) | 0.138 |
| Systolic BP (mmHg) | 130 ± 20 | 127 ± 22 | 0.770 |
| Diastolic BP (mmHg) | 77 ± 11 | 75 ± 12 | 0.775 |
| Heart rate (beats/min) | 64 ± 10 | 64 ± 9 | 0.986 |
| Duration of disease (months) | 35.8 ± 19 | 30.9 ± 40 | 0.746 |
| UPDRS Part 2 (points) | 11.8 ± 5.6 | 8 ± 5.3 | 0.173 |
| UPDRS Part 3 (points) | 21.2 ± 9.4 | 13.6 ± 7.9 | 0.095 |
| H&Y stage | 2 (1.5-3.0) | 2.5 (1.3–3.0) | 0.799 |
| LEDD (mg/day) | 509 ± 237 | 248 ± 269 | 0.0497 |
| Constipation (%) | 46 | 54 | 0.312 |
| CVRR, rest (%) | 3.9 (2.2–4.5) | 2.4 (1.4–2.7) | 0.194 |
| CVRR, deep breath (%) | 2.8 (2.3–3.9) | 2.6 (1.9–4.0) | 0.810 |
| Early H/M ratio | 2.01 ± 0.67 | 1.83 ± 0.43 | 0.515 |
| Delayed H/M ratio | 1.79 ± 0.77 | 1.58 ± 0.68 | 0.516 |
| Orthostatic hypotension (%) | 83 | 17 | 0.064 |
| LF power, supine (ms^2^) | 258 (79–366) | 50 (25–60) | 0.030 |
| HF power, supine (ms^2^) | 117 (42–517) | 41 (14–132) | 0.269 |
| LF/HF ratio, supine | 2.25 (0.78–4.45) | 0.96 (0.55–2.46) | 0.178 |
| LF power, standing (ms^2^) | 84 (47–372) | 86 (18–124) | 0.736 |
| HF power, standing (ms^2^) | 47 (7–440) | 32 (20–114) | 0.736 |
| LF/HF ratio, standing | 4.49 (0.74–13.4) | 2.10 (0.95–6.15) | 0.532 |

Values are expressed as the mean ± SD or median (25%–75% quartile).

Abbreviations: UPDRS, unified Parkinson’s disease rating scale; H&Y, Hoehn and Yahr; LEDD, levodopa equivalent daily dose; LF, low frequency; HF, high frequency; CVRR, coefficient of variation of RR intervals; BP, blood pressure.

Constipation: Participants who had no bowel movements daily or were taking laxatives.


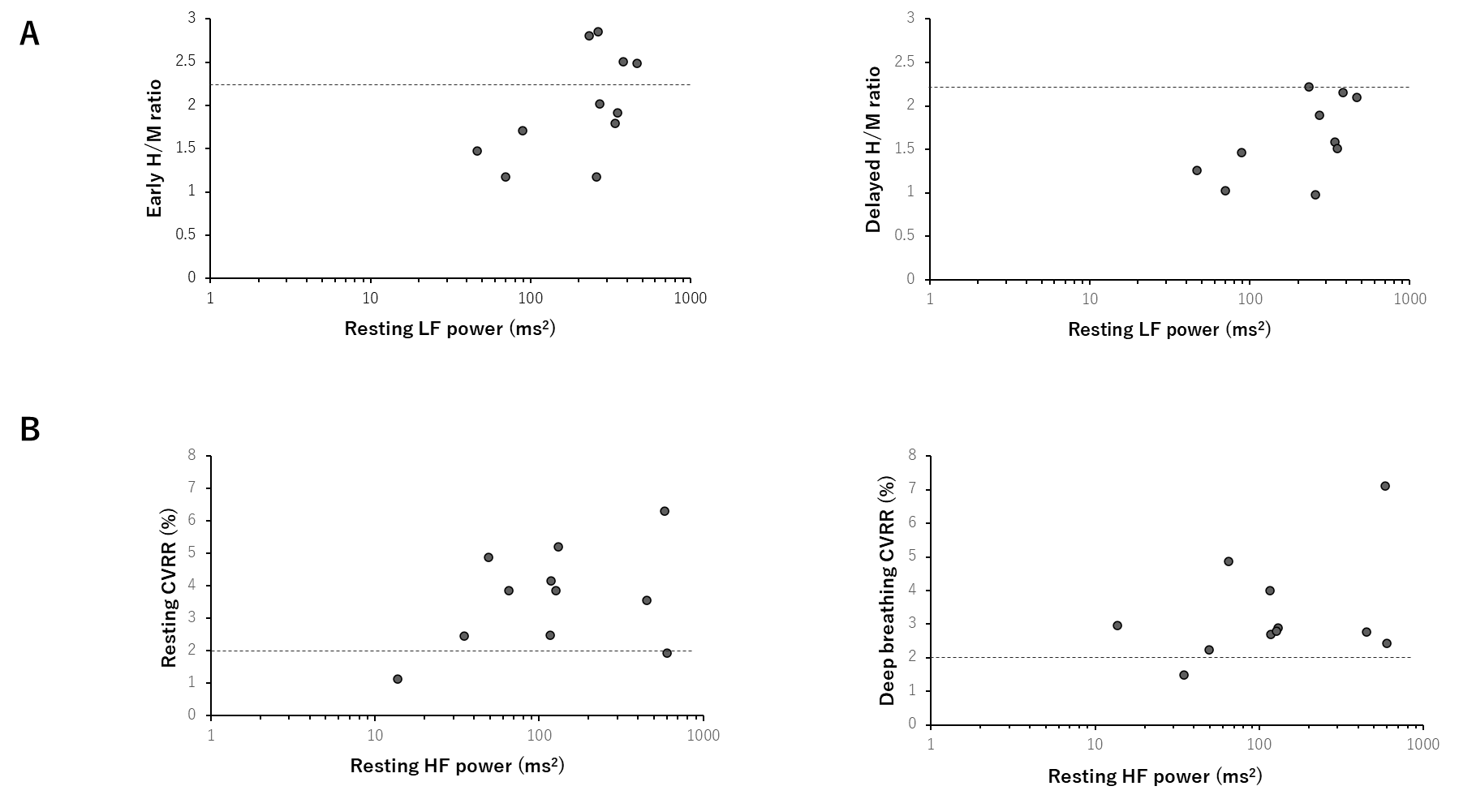


Supplementary Figure 2. Correlations between resting LF power and heart-to-mediastinum (H/M) ratio of ^123^I-meta-iodobenzylguamidine (MIBG) uptake (*A*) and correlations between resting HF power and coefficient variation of RR intervals (CVRR) (*B*) in male patients with PD.

*A*, No correlations was found between H/M ratio and resting LF power in men (early, r=0.58, *p＝*0.10 ; delayed, r= 0.43, *p=*0.24). The dashed line shows the cutoff value for the H/M ratio (2.2). ,*B*, No correlations was found between CVRR and resting HF power in men (rest, r=0.24, *p=*0.54 ; deep breath, r= 0.42, *p=*0.26). The dashed line shows the cutoff value of the CVRR (2.0).


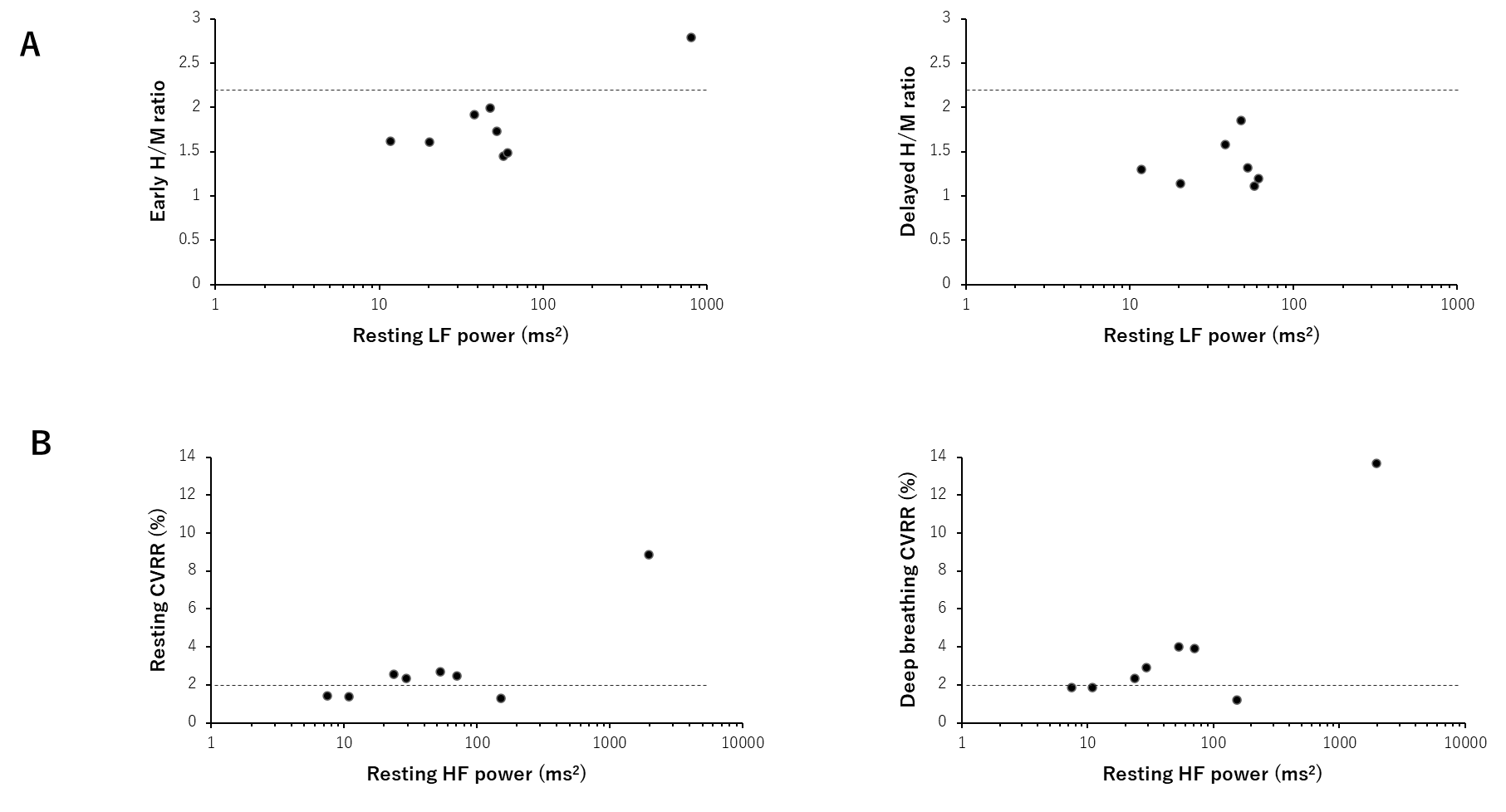


Supplementary Figure 3. Correlations between resting LF power and heart-to-mediastinum (H/M) ratio of ^123^I-meta-iodobenzylguamidine (MIBG) uptake (*A*) and correlations between resting HF power and coefficient variation of RR intervals (CVRR) (*B*) in female patients with PD.

*A*, Significant correlations were found between H/M ratio and resting LF power in women (early, r= 0.89, *p*<0.05 ; delayed, r= 0.93, *p*<0.05). The dashed line shows the cutoff value for the H/M ratio (2.2). ,*B*, Significant correlations were found between CVRR and resting HF power in women (rest, r=0.96, *p*<0.01 ; deep breath, r= 0.84, *p*<0.01). The dashed line shows the cutoff value of the CVRR (2.0).


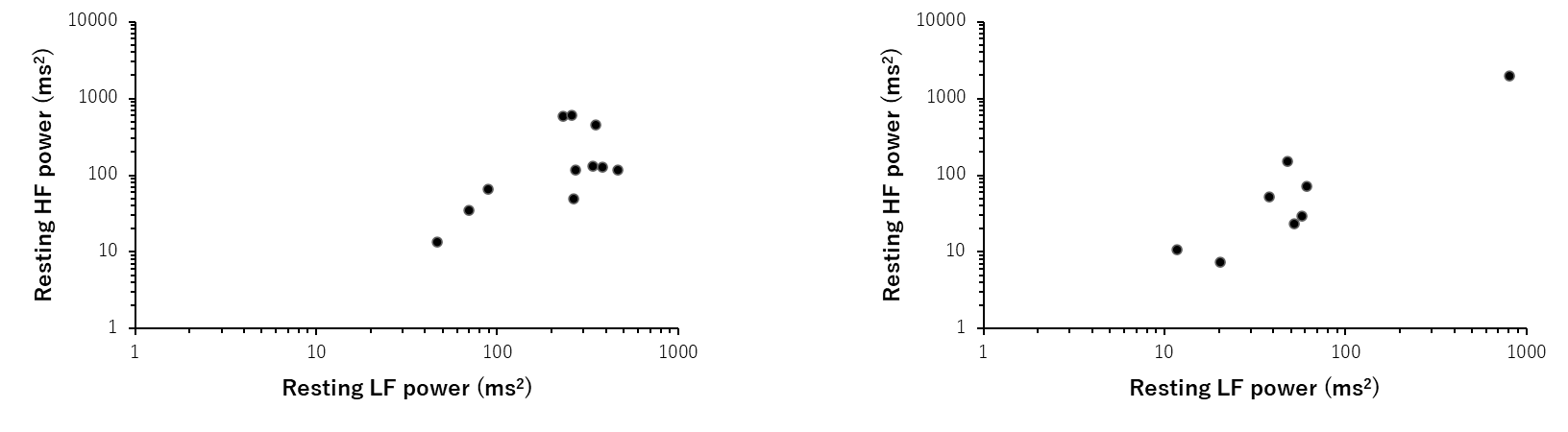


Supplement Figure 4. The correlation between resting LF power and HF power in the patients with PD by gender (*A.* Men, *B.* Women).

*A*, No correlation was observed between resting LF powers and HF powers in men (r=0.29, p=0.44). *B*, A positive correlation was observed between resting LF powers and HF powers in women (r =0.997, p<0.001).
